# Influence of COVID-19 Protocols on the Efficiency of Trauma Theater: Retrospective Observational Study

**DOI:** 10.2196/35805

**Published:** 2022-07-12

**Authors:** Faisal Mohammed, Momin Mohaddis, Manikandar Srinivas Cheruvu, Richard M Morris, Zahra Naim, Sarfraz Khan, Muhammad Babar Mushtaq, Prakash Chandran

**Affiliations:** 1 Warrington Hospital Warrington United Kingdom; 2 Robert Jones and Agnes Hunt Hospital Gobowen United Kingdom; 3 Ysbyty Gwynedd Hospital Bangor United Kingdom; 4 Shadan Institute of Medical Sciences Hyderabad India

**Keywords:** operation theatre, COVID-19, utilization, efficiency, pandemic, health care, trauma management, infection control, health care delivery, trauma theater

## Abstract

**Background:**

The COVID-19 pandemic has influenced health care delivery significantly. Numerous studies have highlighted that trauma theater efficiency has decreased during the COVID-19 pandemic; however, there is limited information as to exactly which stage of the patient theater journey is causing this decreased efficiency and whether efficiency can be improved. In the trauma theater of Warrington Hospital, United Kingdom, we have attempted to maintain trauma theater efficiency despite the requirement for increased infection control.

**Objective:**

The aim of this study was to evaluate the effects of additional COVID-19 infection control protocols on trauma theater efficiency in our center, considering the length of time taken for specific theater events, and to find out whether our interventions were successful in maintaining theater efficiency.

**Methods:**

We compared the efficiency of the trauma theater in a busy unit in December 2019 (pre–COVID-19) and December 2020 (with COVID-19 protocols in place). We collected time logs for different theater events for each patient in December of both years and compared the data.

**Results:**

There was no significant difference in the average number of cases performed per session between the COVID-19 and pre–COVID-19 time periods (*P*=.17). Theater start time was significantly earlier during the COVID-19 period (*P*<.001). There was no significant difference between the two periods in transport time, check-in time, preprocedure time, anesthetic time, and the time between cases (*P*>.05). A significant difference was observed in the check-out time between the two groups in the two time periods, with checking out taking longer during the COVID-19 period (*P*<.001).

**Conclusions:**

Our results show that our theater start times were earlier during the COVID-19 pandemic, and the overall theater efficiency was maintained despite the additional COVID-19 infection control protocols that were in place. These findings suggest that well-planned infection control protocols do not need to impede trauma theater efficiency in certain settings.

## Introduction

There is no doubt the incidence of all acute traumas decreased during the COVID-19 lockdowns. One study demonstrated a decrease of 22.4% in the overall incidence of trauma [1], but the incidence of falls and hip trauma in particular increased compared to the pre–COVID-19 period [2-4]. In December 2020, COVID-19 restrictions were in place across the United Kingdom. In the trauma theater of Warrington Hospital, the infection control protocols were in place from the onset of the pandemic and had become refined. Infection control protocols had a major impact on how surgeries were performed. The patient was brought into the operating room (OR) directly from the ward with no stops in between.

Several studies reported an increase in patient turnaround times with a decrease in theater utilization times, resulting in an overall decrease in theater efficiency during the COVID-19 pandemic due to the additional infection control protocols [5-7].

The aim of this study was to evaluate the effects of additional COVID-19 infection control protocols on trauma theater efficiency in Warrington Hospital, considering the length of time taken for specific theater events, and to find out whether our interventions were successful in maintaining theater efficiency.

## Methods

### Procedure

This study was performed in a busy district general hospital. Because all traumas with an Injury Severity Score >15 goes to a major trauma center in the United Kingdom, the district general hospitals usually handle closed fractures, mostly fragility fractures, including neck of femur or fractures in adults and children that are isolated injuries. We retrospectively analyzed all patients undergoing an operation in the trauma theater of Warrington Hospital, in Warrington, United Kingdom, during December 2019 and December 2020 (ie, when COVID-19 infection control protocols were in place). In our hospital, for all patients, their theater journey events time was recorded live into ORMIS software (version [7.55]; iPath Software). The time log events that were relevant to our study and recorded were “patient sent for,” “patient at reception,” “patient in AR,” “administration of anesthesia start time,” “anesthesia administration complete,” “patient shifts into OR,” “procedure start,” “procedure end,” and “out of OR.”

The above data for all patients having trauma surgery during December 2019 and December 2020 were collected retrospectively and compared. All patients undergoing an operation in the trauma theater were included.

The differences between the two groups with regard to patient flow or pathway are detailed in Table 1. The key difference in patient flow was that the AR and the recovery room were not used during December 2020. During the COVID-19 pandemic period (December 2020), we used an “isolated corridor” for shifting patients from the ward to the theater, where the porters had to clear the whole path of the patient transport to avoid cross-infections. We had to inform the ward and the porters in advance. In the ward, the patient was prepared and kept ready to be shifted; porters arranged the isolated corridor for the transfer. A 15-minute telephone warning was given to the ward.

The patient was brought into the OR directly from the ward with no stops in between. The patients were checked at the entrance of the OR, they were then taken into the OR where the anesthesia was administered, surgery was performed, and the patient was recovered. Prior to COVID-19, the patients were transferred from the ward to the theater complex, the safety checks were performed at the theater reception, patients were then transferred into the AR, where the anesthesia was administered; patients were then taken to the OR, where the surgery was performed; following surgery, the patients were transferred into the recovery room for extubating and recovery from anesthesia.

During the COVID-19 pandemic period (December 2020), personal protective equipment was used by all staff entering the OR. All cases were considered COVID-19 suspect, as we aimed to take every trauma case to theater as early as possible, and there was not enough time for positive results to show up in a good number of patients [8,9]. We chose the month of December 2020 for data analysis as COVID-19 infection protocols had been in place for several months, allowing staff to become sufficiently familiar with them, comparable to the familiarity they had with the pre–COVID-19 protocols a year prior. Further, we chose December because it has a higher volume of trauma operating, with previous studies showing a higher proportion of admitted patients requiring surgery during winter in the United Kingdom [10]. There is added winter pressure on the hospitals in December, and the second COVID-19 lockdown restrictions were in place during December 2020; this made the month ideal for comparison with pre–COVID-19 times.

The calculated time durations for preprocedure time, check-out time, and transport time are detailed in Table 2. The ORMIS software allows for the recording of different events with timed entries. When the theater staff call the ward to send for the patient, “patient sent for” is recorded; and when the patient arrives at the common theater complex, “in suite” time is recorded—this has been termed as the “transport time” in our paper. A member of the theater staff then goes through a checklist with the patient and takes a formal handover from the nurse accompanying the patient. The checklist includes patient identification, confirmation of consent, patient belongings, dentures, nil by mouth status, etc. This is then followed by the patient being shifted into the AR and “in anesthesia room” time is recorded. A World Health Organization (WHO) Surgical Safety Checklist sign-in is done next, followed by preparation and administration of anesthesia. When the anesthetist begins the process, “anesthetist start time” is recorded, followed by “anesthesia ready,” when the patient is anesthetized and ready to be shifted to the OR. Once in the OR, “in OR” time is recorded, the patient is positioned on the operating table, preprocedure imaging is done if needed, and the surgeons scrub. The “procedure start” time is recorded when the WHO time-out is done. The time interval from “in anesthesia room” to “procedure start” defines the “preprocedure time” in our study. “Procedure end time” is recorded when the surgical wound is dressed. This is closely followed by the WHO sign-out phase and shifting of the patient out of the OR when the “out of OR” time is recorded in ORMIS. The time interval between “procedure end” to “out of OR” constitutes the check-out time. In addition, check-in time (ie, “patient at reception” to “patient in AR”) and anesthetic time were also calculated. The time between cases was calculated as the time duration between “out of OR” of the previous case to “in AR” of the next case. The time between cases was not measurable for the last case of the day.

The standard theater functioning time was calculated from 8 AM to 5 PM (for 2 sessions). If the list overran the 2 sessions, then we considered it as a 3-session list in both groups. The statistical analysis was done using IBM-SPSS software (Version [1.0.0.1406]; IBM Corp). The independent *t* test (2-tailed) was applied to test statistical significance between means of unrelated groups. This was preceded by Levene Test for equality of variances. The *t* test was modified if equal variances were not assumed to use unpooled variances and correction of degrees of freedom.

**Table 1:** Comparison of theater events recorded in pre–COVID-19 and during COVID-19 time periods.

Theater events	Time period	
	December 2019 (pre–COVID-19)	December 2020 (during the COVID-19 pandemic)
At theater reception	Ward nurse gives a handover to theater practitioner Safety checklist done by theater practitioner	Theater reception area was not used The handover and checklist were done outside the operating room entrance
In anesthetic room	Signing in Preparation of anesthesia Anesthesia given, including spinal, nerve blocks, arterial access, venous access, and catheterization	Not used (as thoroughfare only)
In operating room	Safety check Patient positioning Pre-op imaging Procedure Shifting out of operating room	Signing in Preparation of anesthesia Anesthesia given, including spinal, nerve blocks, arterial access, venous access, and catheterization Safety check Patient positioning Pre-op imaging Procedure Shifting out of operating room Extubation Wait for 15 minutes after Aerosol generating procedure Recovery from anesthesia and transfer to the ward Cleaning of theater
In recovery room	Extubation Recovery of patient	Not used

**Table 2:** Calculated time durations and their correlation to the time logs in the ORMIS software (version [7.55]; iPath Software) and patient location in 2019 and 2020.

Time duration calculated and the log entry in ORMIS software		Location of patient	
		December 2019	December 2020
**Transport time^a^**			
	“Patient sent for” and “patient at reception”	Patient transported from the ward to the theater reception	Patient transported from the ward to the OR^b^ door
**Preprocedure time^c^**			
	“In anesthesia room”	Patient in AR^d^ (preparation for anesthesia begins after safety check)	In AR (only used as a thoroughfare)
	“Anesthetist start time” (anesthetist begins procedure)	In AR	In OR
	“Anesthetist end time” (patient is anesthetized and ready)	In AR	In OR
	“In OR” (patient shifted to the OR)	In OR	In OR
	“Procedure start”	In OR	In OR
**Check-out time^e^**			
	“Procedure end”	In OR	In OR
	“Out of OR”	Exiting OR	Exiting OR

^a^From when the patient is sent for to when the patient is at the reception or theater front door.

^b^OR: operating room.

^c^From when the patient is in the anesthetic room to the procedure start.

^d^AR: anesthetic room.

^e^From the procedure end to out of the operating room.

### Ethical Considerations

Ethics approval and consent to participate were not required for this study, as the data were collected for quality improvement and as part of an audit.

## Results

A total of 76 patients underwent an operation in our trauma theater in December 2019, and a total of 68 patients in December 2020. In December 2019, the 76 cases were operated in 68 sessions, at an average of 1.11 cases per session, while in December 2020, the 68 cases were operated in 66 sessions, the average being 1.03 cases per session (*P*=.17, *t* test). The average time when the first case entered the OR in December 2019 was 10:39 AM, and in December 2020, it was 9:36 AM *(P*<.001, *t* test). The average time of the last case out of the OR in December 2019 was 4 PM and in December 2020 was 5 PM *(P=*.09, *t* test).

There was no significant difference between the two groups in the time log calculation of the transport time, check-in time, preprocedure time, anesthetic time, and the time between cases. However, a significant difference was observed in the check-out time between the two groups (Table 3).

**Table 3:** Comparison of the length of time for various theater events between pre–COVID-19 and during COVID-19 time periods.

Theater event time duration	Length of time (min:s)		*P* values (*t* test)
	December 2019	December 2020	
Transport time	20:51	19:56	.74
Check-in time	07:56	08:56	.63
Preprocedure time	45:12	46:36	.63
Anesthetic time	20:09	23:44	.10
Check-out time	10:21	20:51	<.001
Time between cases	46:30	46:48	.41

## Discussion

### Overview

The principal findings of our study showed that there was no statistical difference in the transport time, check-in time, preprocedure time, anesthetic time, and time between cases of the two groups, even though infection control protocols were changed during December 2020.

Our study found that there was no significant difference between the COVID-19 and pre–COVID-19 periods with regard to theater efficiency, unlike several other studies [5-7]. We observed a similar number of operations being performed per theater session across both groups, with the only significant difference between groups occurring in the “procedure start” time and the length of time for check-out after a procedure.

### Comparison With Prior Work

We observed a decrease in the number of cases operated during the pandemic period, which is similar to the observations made by Andreata et al [11], who found a decrease in the number of surgeries done during the COVID-19 lockdown compared to a similar time period before the pandemic.

In our study, the timing of starting the first case significantly improved during December 2020. This is in contrast to the findings by Khadabadi et al [7], who found that their start time was significantly higher during the COVID-19 pandemic, and 94.2% of their lists began late in 2020. The start time of the first case is one of the main measures of theater efficiency [12,13]. Delay in starting the first case could cause delays in the whole list as a downstream effect [14]. In pre–COVID-19 times, the trauma list was populated at 8 AM every day, followed by patient assessment by the surgical and anesthetic team and a huddle in the theater. There was a single anesthetist allocated to the trauma theater, who would assess the cases listed for the theater and then attend the huddle. During December 2020 (ie, COVID-19 pandemic), the trauma list was populated at 8 AM every day. There was an extra anesthetist available, as elective operating was suspended. The anesthetist assessed the first patient and was available in the theater for the huddle, while the second anesthetist continued to assess the remaining patients on the list. The ward rounds were also shorter for the surgical team due to fewer admissions. We believe these changes contributed significantly to the earlier start times in December 2020.

Transport of patients from ward to theater was found to be a cause of significant decrease in theater productivity in a few studies [15,16]. We observed no statistically significant difference in the transport times between the two groups. For shifting of patients, we used an “isolated corridor,” where the porters had to clear the whole path of patient transport to avoid cross-infections; a 15-minute telephone warning to the porters and the ward helped to keep the patient and corridor ready. This is similar to a study by Ang et al [17] that has shown that the availability of dedicated porters in the theater can improve transport times.

Delay in sending for the patient was also a significant factor causing decreased theater efficiency in a study by Ang et al [17]; however, due to the “isolated corridor” policy, we had to inform the porters earlier, so they were ready in time to shift the patient; a 15-minute telephone warning was also given to the ward, so that the patient was ready when the porters arrived.

Several studies have found a significant increase in their theater turnaround times (ie, time between cases) during the COVID-19 pandemic [5-7]. In our trauma theater, the patient was intubated and extubated in the OR itself, giving a window of 15 minutes after any aerosol generating procedure, with minimal necessary staffing in the room during that time; the doors were not to be opened in accordance with infection control protocols. The OR and AR underwent a “deep clean” and surfaces were wiped with the viricidal wipes after every case. The trolleys and other instruments that were not necessary were shifted to another room apart from the OR. Despite these additional steps, we observed no statistically significant difference in our time between cases in the two groups. As elective procedures were paused, the staff from elective surgery theaters were used at the trauma theater. This made additional staff available outside the OR to do the cleaning. There was also one extra operating department practitioner in the theater who helped in the arrangement of the trolleys and doing pre-op checks as soon as the next patient arrived, while the current patient was recovered.

Although the requirement to wear personal protective equipment within the AR or OR can add to the turnaround time and has been found to decrease theater efficiency [18], the staff within our hospital made sure the time for donning and doffing did not delay any proceedings. Preparedness, a dedicated cleaning team, and sending for the next patient as soon as cleaning began, in addition to the improvement in transport arrangements helped to maintain our time between cases. Daniel Fletcher et al [19] have observed that the theater time between cases (ie, turnaround time) can be improved by the introduction of a 15-minute warning to the preoperative rooms, performing the check-in process in the preoperative rooms rather than in the theater, sending for the patient prior to the completion of theater cleaning and finally, a 5-minute warning given to the theater cleaning staff. They have shown a 45% reduction in mean turnaround times with these measures [19].

Our study found no difference in the average anesthetic time and pre-procedure time, similar to the study by Mercer et al [5]. Regional anesthesia techniques and anesthesia delivered by senior doctors (ie, consultants and senior registrars) were thought to decrease anesthetic time [5]. Our observations were similar, with largely the consultants themselves performing the procedures, and trainees redistributed to care for COVID-19 patients in the wards. Preparedness of the team and a preference for regional anesthesia, thus avoiding the 15-minute aerosol generating procedure downtime, also helped to reduce the anesthetic time.

We observed a significant difference in check-out times, as the patient spent more time in the OR during the COVID-19 period. This was due to the patient recovering in the OR and subsequently being transferred to the ward directly from the OR.

The overall theater efficiency of our study is different compared to other studies, which have shown a significant decrease in theater efficiency [5-7,9] at varying time events during the patient journey.

### Strengths and Limitations

Our study has certain strengths. It presents the systematic analysis of a patient journey through theater, examining every step and the causes of delays. We have discussed in detail how these delays were overcome without compromising on infection control. We believe our study would help in effective theater management and in turn, reducing the waiting lists.

The study has certain limitations, including the small sample size and clubbing of weekdays and weekends together in data analysis. Nevertheless, the strength of this study lies in the calculation of all time durations involved in the patient journey, which we have not encountered in such detail in any other study during the pandemic. We have not considered the comorbidities of the patients, which might indirectly and insignificantly affect the preprocedure times.

### Future Directions

Further research is needed to calculate the costs of the above-mentioned interventions and how they apply to an elective surgery setting, so that the waiting lists can be reduced by efficient theater management. This study could be used as a template to further investigate theater efficiency in elective surgery theaters. It could also serve as a baseline for a quality improvement project that could apply these interventions and measure the effect of each. The study could also be a helpful guide in making theaters cost-effective.

### Conclusions

Early theater start time, organized patient transport, a 15-minutes prewarning to the ward and the porters, checking patients at the theater entrance, availability of senior anesthetists, preference to use regional anesthesia, and the presence of additional staff can help maintain theater efficiency even with infection control protocols in place during the COVID-19 pandemic. Effective theater management will also have implications for the waiting lists.

## Abbreviations

AR: anesthetic room
OR: operating room
WHO: World Health Organization

## Data Availability

Data are available upon reasonable request, through contacting the corresponding author via email.
